# Extreme hyperleukocytosis in a pediatric T-ALL patient with a rare translocation, t(7;19)(q35;p13), and submicroscopic deletions at 4q25, 7q33 and 10q23^☆^

**DOI:** 10.1016/j.lrr.2013.09.004

**Published:** 2013-11-01

**Authors:** Christopher Veigaard, Anni Aggerholm, Henrik Hasle, Eigil Kjeldsen

**Affiliations:** a Hemodiagnostic Laboratory, Department of Hematology, Aarhus University Hospital, Tage-Hansens Gade 2, Ent. 4A, 8000 Aarhus C, Denmark; b Department of Pediatrics, Aarhus University Hospital, Brendstrupgaardsvej 100, 8200 Aarhus N, Denmark

**Keywords:** Acute T-lymphoblastic leukemia, Hyperleukocytosis, t(7;19), *LYL*1, aCGH analysis

## Abstract

Although childhood T-cell acute lymphoblastic leukemia (T-ALL) is a high-risk disease the outcome can vary considerably. The varying outcomes suggest that unrecognized factors may contribute to disease progression. We report on a 2-year-old T-ALL patient presenting with a very short history of constipation and extreme hyperleukocytosis (WBC 882×10^9^/L). In her leukemic cells we detected the very rare translocation t(7;19)(q35;p13) and *LYL*1 overexpression. Additionally, we detected submicroscopic deletions at 4q25, 7q33 and 10q23 by oligo-aCGH analysis. We suggest that *LYL*1 overexpression contributed to the leukemic state and propose that the observed microdeletions may have influenced to the rapid disease progression.

## 1 Introduction

T-ALL is a heterogeneous disease, accounting for 10–15% of childhood ALLs, reviewed in [1]. The disease is less common in young children, and presents with a median WBC count of 75×10^9^/L. Cytogenetic abnormalities are seen in approximately 50% of T-ALL patients. Cryptic translocations and deletions, involving e.g. *TLX*3 and *TAL*1, can be detected by FISH. Translocations involving the T-cell receptor loci are found in approximately 35% of T-ALL. Aberrant expression of one or more transcription factors, such as for example *TAL*1, *TAL*2, *LYL*1, *OLIG*2, *MYC* and *LMO*1/2, is a critical component of the molecular pathogenesis of T-ALL. Activating NOTCH1 mutations are present in the majority of T-ALL cases.

In contrast to pre-B-ALL the genomic characterization of T-ALLs has limited prognostic impact. Thus, further improvement in treatment choice and outcome may rely on improved characterization of the cytogenetic and molecular events involved in this high-risk malignancy.

We characterized the genomic complement in a pediatric T-ALL case with a rapid and aggressive clinical course albeit discrete pre-diagnostic symptoms. We detected the very rare t(7;19)(q35;p13), additional submicroscopic deletions at 4q25, 7q33 and 10q23, and a marked overexpression of *LYL*1.

## 2 Materials and methods

G-banding, FISH analyses and high resolution aCGH analysis using CytoChip Cancer 180K microarrays (BlueGnome, Cambridge, UK) were done on bone marrow cells at diagnosis as described [2]. The following FISH probes were used: 24XCyte (MetaSystems, Altlussheim, Germany), whole chromosome painting probe chromosome 19 (Kreatech Diagnostics, Amsterdam, Netherlands), *TRB*, *STIL-TAL*1, *TCF*3, *MYC* (Dako, Glostrup, Denmark), *MLL* (Abbott Laboratories, Illinois, USA), BlueFish probes RP11-148D11 (19p13.13) and RP11-356L15 (19p13.13) (BlueGnome) and BAC probes RP11-840M18 (4q25), RP11-765A17 (7q33), and RP11-79A15 (10q23.2q23.31) (EmpireGenomics, New York, USA), centromeric probes for chromosomes 4 (D4Z1), 7 (D7Z1), and 10 (D10Z1) (Kreatech Diagnostics). Reference genome was hg18. Relative *LYL*1 gene expression was investigated with TaqMan technology as described [3].

## 3 Clinical description

A previously healthy 2-year-old girl presented with a 2-week history of constipation and sniveling. The day before admission she developed fever to 38.4 °C. On admission she was relatively well despite thrombocytopenia (platelets=44×10^9^/L) and anemia (Hgb=4.4 mM). WBC count was 882×10^9^/L, increasing to 938×10^9^/L within 12 h. At admission petechiae were noted without signs of bleeding. She became unconscious 12 h later and needed ventilator. CT scan showed a large intracerebral bleeding. Despite optimal supportive care and a favorable response to corticosteroids with WBC of 121×10^9^/L on day 3, new intracranial bleedings occurred. The patient incarcerated and died on day 4 from admission.

## 4 Results

**Fig. 1:**
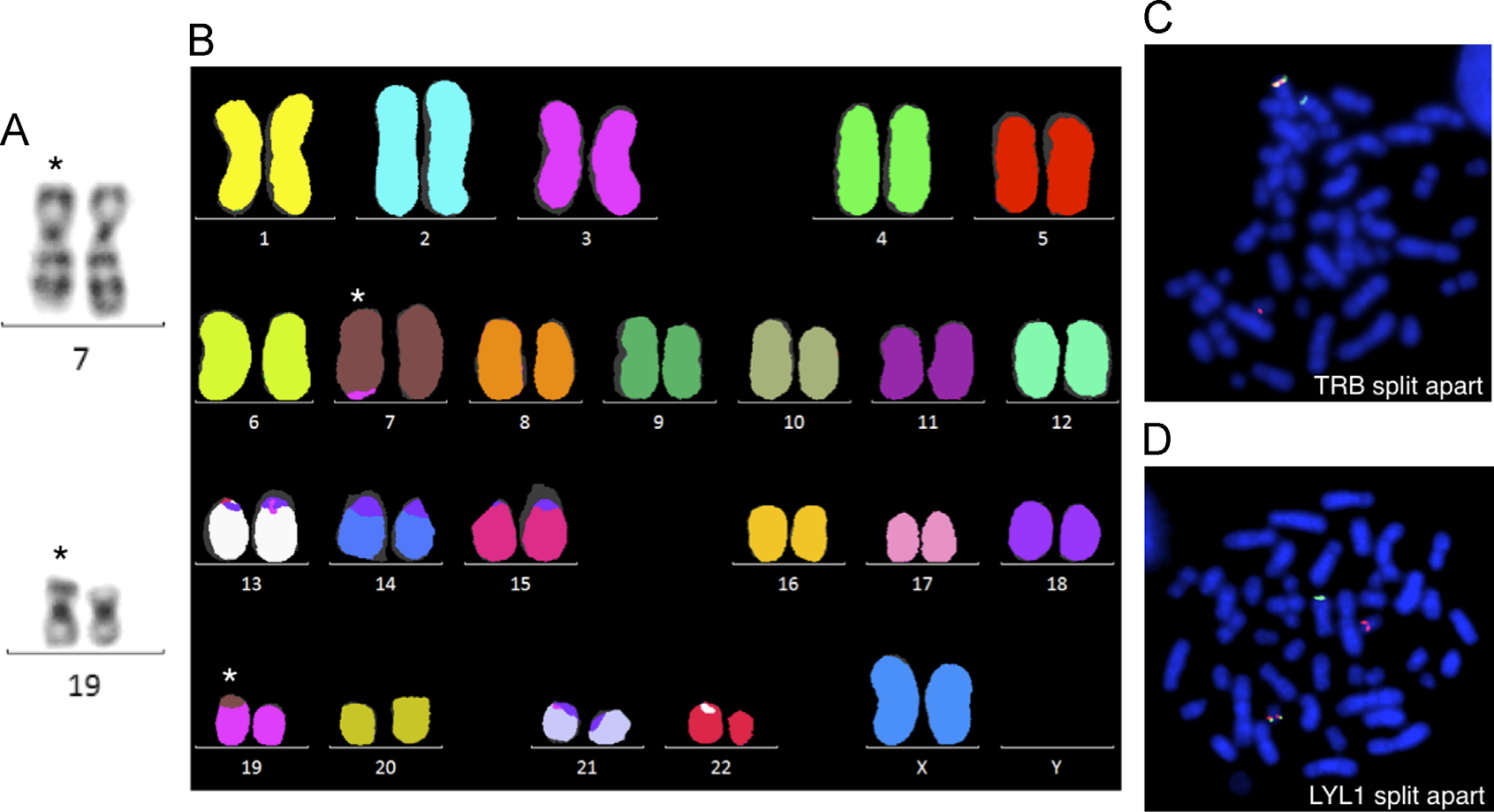
Cytogenetic analyses: (A) partial G-banded karyogram showing chromosome pairs 7 and 19; asterisk indicates translocated chromosomes; (B) 24-color mFISH karyogram; asterisk indicates translocated chromosomes; (C) metaphase FISH with a commercial *TRB* split apart probe (7q35) and (D) metaphase FISH with a *LYL*1 split apart probe, RP11-148D11 (green) and RP11-356L15 (red) at 19.13.13. (For interpretation of the references to color in this figure legend, the reader is referred to the web version of this article.)

**Fig. 2:**
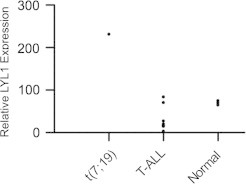
Relative expression of *LYL*1 in our present case, six T-ALL patients without t(7;19) and four normal donors.

**Fig. 3:**
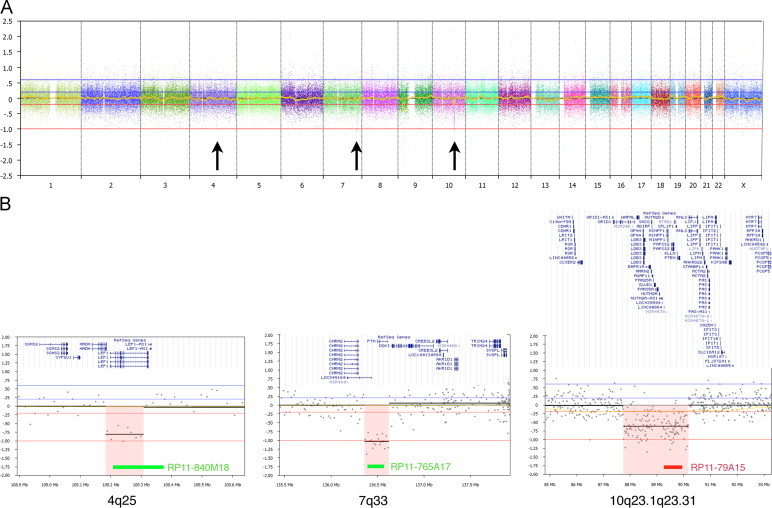
High-resolution oligo-based aCGH analysis: (A) whole genome profile, arrows indicate positions of deletion; and (B) zoom view of deleted region at 4q25 (left panel), 7q33 (middle panel), and 10q23.1q23.31 (right panel). The green and red bars indicate the sizes and approximate positions of the BAC probes used for confirmation (see Fig. 4). Insets are RefSeq genes from indicated regions according to UCSC, hg18. (For interpretation of the references to color in this figure legend, the reader is referred to the web version of this article.)

**Fig. 4:**
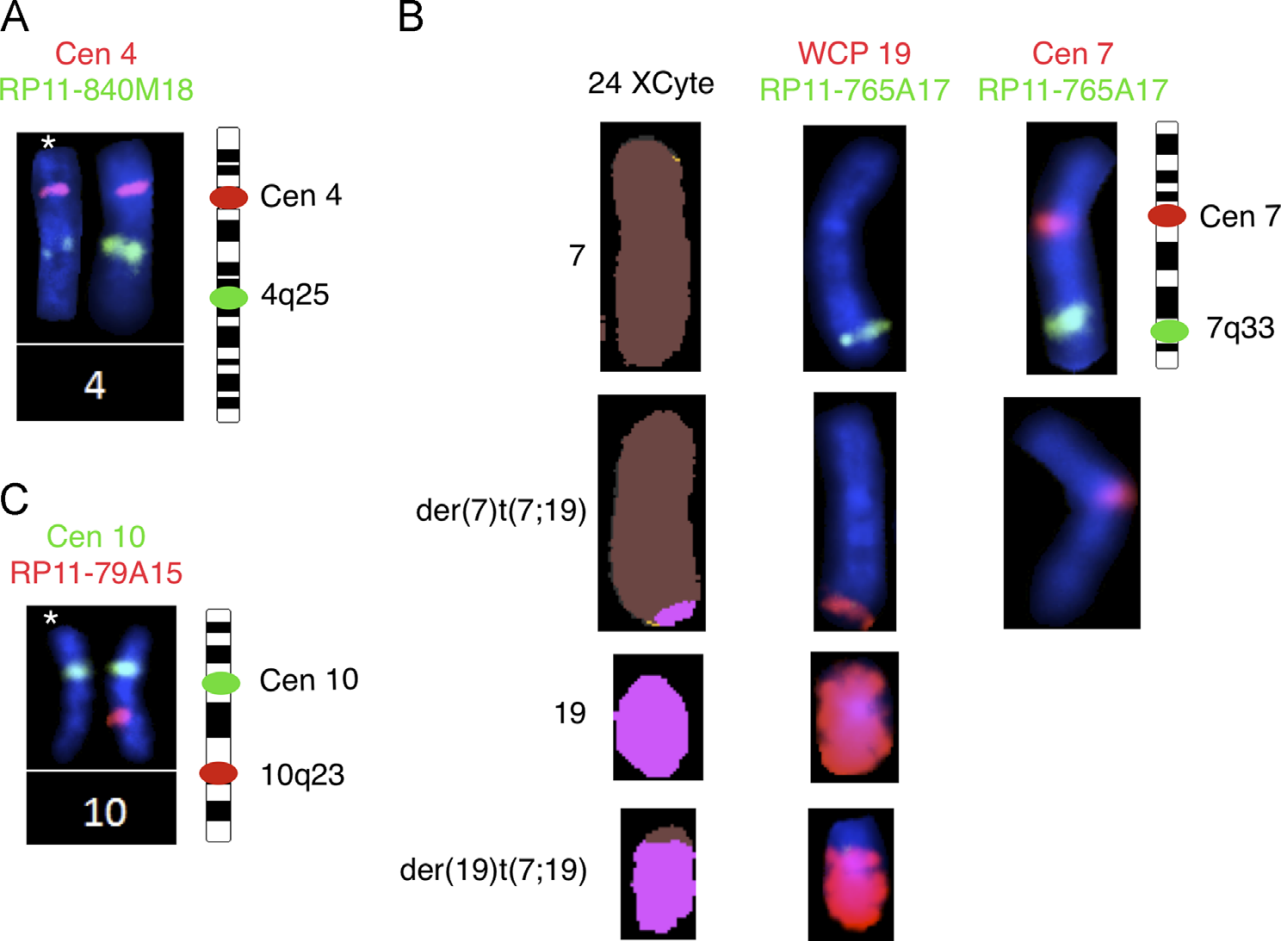
FISH analyses confirm the deletions detected by aCGH analysis. Aberrant chromosomes are indicated by asterisks or by text: (A) partial karyogram showing chromosome pair 4 after metaphase FISH with centromeric probe D4Z1 and RP11-840M18. This BAC probe is the available best fitting probe for confirmation in this region, and it is noted that it gives a weak signal on the aberrant chromosome 4 and a very strong signal on the normal chromosome 4. This observation is in agreement with the size and position of the probe relative to the size and position of the deleted region on chromosome 4 as detected by aCGH analysis. (B) Partial karyogram showing chromosome pairs 7 and 19 after 24-color karyotyping (left panel); metaphase FISH with RP11-765A17 and whole chromosome painting probe for chromosome 19 (middle panel); and metaphase FISH with RP11-765A17 and centromeric probe D7Z1 (right panel). (C) Partial karyogram showing chromosome pair 10 after metaphase FISH with RP11-79A15 and centromeric probe D10Z1.

The leukemic cells showed 46,XX,t(7;19)(q35;p13)[23]/46,XX[2] (Fig. 1A and B). FISH analyses of common T-ALL associated aberrations (*STIL-TAL*1, *MLL*, *TCF*3 and *MYC*) were negative. FISH with a commercial split-apart probe for the *TRB* gene at 7q35 showed 94% positivity (Fig. 1C). The only previously known translocation partner for *TRB* at chromosome 19 band p13 is *LYL*1. Since no commercial FISH kit for *LYL*1 locus is available, we designed a split probe assay using two BAC probes (RP11-148D11 and RP11-356L15), located approximately 0.02 Mb from the *LYL*1 locus on each side and established that the second breakpoint was located close to the *LYL*1 locus between the two probes (Fig. 1D). The expression of *LYL*1 in her leukemic cells was markedly increased compared to controls (Fig. 2). Three additional submicroscopic genomic changes were detected by high-resolution oligo-aCGH analysis at 4q25 (pos. 109,182,833–109,307,857), 7q33 (pos. 136,358,795–136,622,676), and at 10q23.1q23.31 (pos. 87,745,813–90,181,623) (Fig. 3). For confirmation we used the BAC probes RP11-840M18 (4q25), RP11-765A17 (7q33), and RP11-79A15 (10q23.2q23.31) with positions as indicated in Fig. 3. Hybridizations with these probes confirmed all deletions (Fig. 4). In addition, we found that approximately 90% of the nuclei contained each of the deletions, which indicates that there were no subclones. Interestingly, we could show that the observed deletion at 7q33 is on the same homolog as the translocation (Fig. 4B). In silico analysis indicates that the genomic distance between these two chromosomal aberrations is approximately 5.9 Mb. It is a possibility that the translocation t(7;19)(q35;p13) and the del(7)(q33q33) occurred in the same genetic event, but from these experiments it can, however, not be established that this is the fact.

## 5 Discussion

Hyperleukocytosis is arbitrary defined as WBC count greater than 100×10^9^/L. The critical WBC count seems different in different leukemias. In patients with AML a leukocyte count of 50×10^9^/L can cause severe symptoms, while patients with CLL can remain asymptomatic even with WBC counts greater than 500×10^9^/L. Hyperleukocytosis is associated with a high risk of severe complications and mortality [4]. Leukostasis with intracranial bleeding is especially frequent in AML with M4 or M5 morphology but may also occur in ALL with extreme leukocytosis like in our patient. Hyperleukocytosis has significant prognostic implications. The prognostic impact of a high WBC count in B-ALL is greater than in T-ALL. An association of hyperleukocytosis with specific subtypes of the leukemia has been observed [5]. It was hypothesized that hyperleukocytosis might be an expression of a molecular change, and that the molecular aberration itself is responsible for the poor prognosis rather than the actual WBC count.

The t(7;19)(q35;p13) is very rare and has only been described in two other T-ALL cases (Table 1). Both cases had hyperleukocytosis, although not to the same extent as in our patient. In the second of reported cases the t(7;19) was cryptic, and presented with an additional subclone containing trisomy 8. It was the sole cytogenetic abnormality in the first published case as well as in ours. Only one patient is still alive. In both cases where the patients died the disease course was rapid. LYL1 was overexpressed in the second case as well as in our case. The LYL1 expression was not examined in the first case.

*LYL*1 belongs to the basic helix-loop-helix (bHLH) transcription factor family, which plays important roles in a variety of developmental processes, including hematopoiesis [6]. The biological function of LYL1 is largely unknown, but its expression is restricted to hematopoietic cells, including myelocytes, erythrocytes and B-lymphocytes in adults. Ectopic expression of LYL1 has been observed in a fraction of human T-ALL and an oncogenic effect of LYL1 was demonstrated in LYL1 transgenic mice. The expression of LYL1 can be deregulated by at least two mechanisms, either because of a translocation or because of a microdeletion [7]. While *LYL*1 overexpression has been linked to suboptimal treatment responses [7] it is unknown how or if this is associated with outcome.

It is becoming more evident that chromosomal abnormalities must act in concert with other genetic lesions to induce overt leukemia. In one of the reported t(7;19) cases an additional submicroscopic deletion near *LMO*2 was found, which coincided with elevated expression of this gene [8]. In our case we detected three additional submicroscopic deletions by aCGH analysis. Although several genes reside within these regions it is of particular interest that the deletions embrace *LEF*1 and *PTEN* at 4q25 and 10q23, respectively. Deletions and inactivating mutations in the LEF1 gene have been reported in pediatric T-ALL [9]. Loss of *PTEN* has been proposed to modulate the aggressiveness of T-ALL and been associated with poor treatment response [10].

Although more cases are needed to disclose the underlying biological mechanisms responsible for the extreme hyperleukocytosis observed in our T-ALL pediatric case with t(7;19)(q35;p13) and additional submicroscopic deletions at 4q25, 7q33 and 10q23, we cannot rule out that deletions of *PTEN* or *LEF*1 genes or other genes in the deleted regions may have influenced to the aggressive course in concert with the elevated *LYL*1 expression.

## Figures and Tables

**Table 1:** Summary of published T-ALL cases with t(7;19)(q35;p13) and present case.

**Reference**	Smith et al. [11]	Homminga et al. [8]	Present case
**Age (yr)**	19	7	2
**Gender**	Male	Male	Female
**WBC (×10**^**9**^ **L**^**−1**^**)**	231	119^a^	882
**Platelets (×10**^**9**^ **L**^**−1**^**)**	373	No information	44
**Immunophenotype**	CD1−, CD3+, CD4+, CD8+, CD34−	CD1−, CD2+, CD3−, CD4+, CD5−, CD7+, CD8+	CD2+, CD3−, CD4+, CD5+, CD7+, CD8+
**Cytogenetics**^b^	46,XY,t(7;19)(q35;p13)	47,XY,+8[6]/46,XX[7], Cryptic t(7;19)(q35;p13) revealed, by FISH	46,XX,t(7;19)(q35;p13)[23]/46,XX[2]
**LYL1 expression**	n.d.	Overexpression	Overexpression
**Outcome**	Died, 6 mo after diagnosis	Alive, 36 mo+ after diagnosis^a^	Died, 4 days after diagnosis

a Personal communication by J.P. Meijerink.

b The breakpoints of the t(7;19) has been assigned according to current band designation of involved genes.
